# Surgical confidence and competence among US veterinary students after a high‐volume sterilisation campaign in rural Mexico

**DOI:** 10.1002/vro2.70025

**Published:** 2026-02-15

**Authors:** Guillermo Arcega Castillo, Rachael Schulte, Melinda J. Wilkins

**Affiliations:** ^1^ Center for Animal Health and Food Safety College of Veterinary Medicine University of Minnesota St. Paul Minnesota USA; ^2^ Department of Veterinary Population Medicine College of Veterinary Medicine University of Minnesota St. Paul Minnesota USA

**Keywords:** experiential learning, soft‐tissue surgery, veterinary surgical education

## Abstract

**Objectives:**

To evaluate whether participation in a high‐volume sterilisation campaign increases veterinary students’ confidence in surgical and anaesthetic skills. We hypothesised that the intervention would improve confidence across key domains and lead to satisfactory technical competence.

**Design:**

Prospective, observational pre–post‐intervention study.

**Setting:**

Field‐based surgical campaign conducted over 10 days in eight rural communities in coastal Oaxaca, Mexico.

**Participants:**

Thirteen fourth‐year veterinary students from two US universities volunteered to participate. Inclusion required students to be in their final year of training; all the students completed the study.

**Interventions:**

Participants engaged in a 10‐day sterilisation campaign, assisting in pre‐, intra‐ and post‐operative care under the supervision of licensed veterinarians. Surgical procedures focused on ovariohysterectomy and orchiectomy in dogs and cats.

**Primary and secondary outcome measures:**

The primary outcome was change in self‐reported confidence across 11 surgical domains, measured pre‐ and post‐intervention using a 0–10 Likert scale. The secondary outcome was technical competence, which was assessed on the final day using a modified Objective Structured Assessment of Technical Skills rubric covering five domains.

**Results:**

Significant increases in confidence were observed in 10 of 11 domains (Wilcoxon *p* < 0.05), with large effect sizes (*r* > 0.5). The highest effect was for ligating blood vessels (*r* = 0.877). Objective Structured Assessment of Technical Skills scores showed consistent proficiency, with domain means ranging from 3.58 to 3.94, and the strongest performance in instrument handling.

**Conclusions:**

Field sterilisation participation increased students’ surgical confidence and end‐of‐campaign competence, but single‐site uncontrolled sample and self‐report limit generalisability; larger multisite controlled studies are needed objectively.

## INTRODUCTION

Developing confidence in surgical skills is a critical component of veterinary education, yet many students often report a lack of confidence and doubt their abilities in procedures such as canine and feline ovariohysterectomy and orchiectomy.^1^, ^2^ Students often feel unprepared to complete surgeries efficiently or avoid complications such as post‐operative bleeding or ovarian remnants.^3^, ^4^ Inadequate hands‐on experience can leave students underprepared, contributing to elevated stress levels that may impair judgement, slow intra‐operative decision making and increase the risk of errors.^5^, ^6^, ^7^ This stress not only compromises patient outcomes but also affects the student's learning and mental wellbeing.^5^, ^6^, ^7^ Moreover, the expectation that new graduates should competently perform routine surgeries, coupled with employers’ assumptions about core surgical proficiency, can be overwhelming.^8^, ^9^, ^10^, ^11^, ^12^ Although the surgical technique is the primary focus of most training models, perioperative anaesthesia support involves distinct competencies and is commonly integrated into surgical learning experiences, particularly in high‐volume settings.

Beyond simple exposure, experiential learning theory suggests that skill develops through iterative cycles of doing, reflecting, conceptualising and reapplying learning in practice.^13^ High‐volume sterilisation campaigns provide concrete experience and repeated opportunities for feedback and refinement, aligning with this model and offering conditions that can foster confidence and core surgical competence.^13^

To address these gaps in surgical training and experiential exposure, training models that provide meaningful, hands‐on surgical experience are essential. The Mazunte Project, established in 2001 in coastal Oaxaca, Mexico, is one such model.^14^ Originally designed to reduce stray dog populations threatening endangered sea turtle nests, the programme also sterilises community cats as part of a broader One Health approach to companion‒animal population management. It also serves as a service‐learning campaign where US veterinary students and licensed veterinarians perform high‐volume sterilisations in rural, low‐resource settings. This environment offers an opportunity to develop core competencies in anaesthesia, surgical flow and tissue handling through intensive hands‐on practice.

To better understand the educational impact of such experiential learning, this study aimed to evaluate whether participation in the Mazunte campaign increased self‐reported confidence in core surgical skills, with perioperative anaesthesia support treated as a complementary secondary component. We also assessed final technical competence using a modified Objective Structured Assessment of Technical Skills (OSATS) rubric. We hypothesised that the campaign would result in increased confidence across key surgical domains and that students would demonstrate satisfactory technical competence by the end of the training.

## MATERIALS AND METHODS

### Study design and setting

We conducted a pre–post‐survey study with OSATS assessment involving US veterinary students as part of the Mazunte Project's annual high‐volume sterilisation campaign (2‒13 January 2025) across eight rural communities near Mazunte, Oaxaca. These sites were selected due to high stray dog populations, proximity to endangered sea turtle nesting areas, and lack of local surgical services. Eligible participants were final fourth‐year US veterinary students who volunteered. Verbal informed consent was obtained from all students prior to participation in the study. This study was reported in accordance with the Strengthening the Reporting of Observational Studies in Epidemiology guidelines.

A structured on‐site orientation was delivered before surgical encounters, including a review of field standard operating procedures (e.g., patient intake, aseptic preparation, anaesthetic monitoring, analgesia and post‐operative recovery/discharge instructions) and live demonstration of the first campaign surgeries by experienced, licensed surgeons. These procedures followed the Mazunte Project's established campaign workflow and surgical training approach, which is implemented consistently across annual field campaigns. Feedback from licensed veterinarians was provided at the start of each day (briefing), during procedures (real‐time coaching), immediately after each surgery (targeted technical points) and at the end‐of‐day debrief to consolidate learning and plan individual objectives for the following day. During procedures, students received continuous chairside mentorship, initially assisting and then progressing to primary surgeon as competency was demonstrated. The typical staffing ratio was three licensed veterinarians for every five students. All student‐performed procedures were conducted under the direct supervision of veterinarians licensed in the United States (US) or Mexico, with informed owner consent. The team included a board‐certified veterinary specialist recognised by the American College of Veterinary Surgeons (ACVS) and collaborators from the Community Spay Neuter Initiative Partnership.

The Mazunte Project's annual campaign sterilises both dogs and cats; students participated in these procedures and gained practical experience with both species relevant to core surgical training. The procedures included ovariohysterectomy in females and orchiectomy in males, all performed under the direct supervision of licensed veterinarians; anaesthesia‐related tasks (dose calculations) were a secondary component of participation.

Animals received a brief pre‐anaesthetic evaluation; cases unsuitable for field anaesthesia were deferred and referred. The field anaesthetic protocol used xylazine (xylazine injectable solution) for premedication and tiletamine–zolazepam (Telazol) for induction with supplemental dosing as needed for surgical duration; atipamezole (Antisedan) was used to reverse alpha‐2 effects during recovery when indicated. Local anaesthesia was incorporated for all procedures: lidocaine (lidocaine HCl injectable solution) intratesticular for castrations, and bupivacaine (bupivacaine HCl injectable solution) incisional/line blocks for ovariohysterectomies. Non‐steroidal anti‐inflammatory drugs were administered perioperatively for analgesia per protocol (carprofen; Rimadyl) in dogs and meloxicam (Melodex) in cats. Standard asepsis (clip, scrub, sterile drape, sterile instruments and gloves) was followed. Physiological monitoring included continuous clinical observation (mucous membrane colour, palpebral reflex and jaw tone) with heart/respiratory rate tracking. Patients were monitored through recovery to stable ambulation (dogs) or full responsiveness (cats).

For female dogs and cats, ovariohysterectomy was performed via a ventral midline approach. The standard steps included midline laparotomy, ovarian pedicle isolation with a three‐clamp technique, pedicle ligation, uterine body ligation and routine three‐layer closure as appropriate for species and body size. For male dogs, prescrotal orchidectomy was the standard approach using a closed technique. For male cats, scrotal orchidectomy was performed using a closed technique with spermatic cord autoligation. Post‐operatively, analgesia was provided via local blocks for all procedures and systemic medications per the protocol. Incisions were inspected prior to discharge, and post‐operative instructions were provided to pet owners in multiple formats—verbal counselling, printed handouts and posted signage that owners could photograph; immediate adverse events were managed on‐site.

Although the research focused on students rather than animals, the Mazunte Project operated with a strong commitment to animal welfare and ethical practice, adhering to the highest standards feasible in the field consistent with published veterinary medical care guidelines for ovariohysterectomy and orchiectomy programmes.^15^ Beyond Institutional Review Board (IRB) oversight of the student survey, the sterilisation campaign itself was conducted under programmatic oversight in collaboration with local partners and community leaders in Mexico, with advance site coordination and owner‐facing communications to support community impact considerations and transparency.

### Data collection

The ‘Veterinary Student Confidence Self‐Assessment’ (Supporting Information S1) was administered on days 1 and 10 using a 0–10 Likert scale. A 10‐point format was chosen to provide greater granularity and sensitivity to change in a small sample and to reduce ceiling effects relative to a five‐point scale.^16^ The self‐assessment was developed by the study team for this project. Items reflected core steps in canine and feline sterilisation workflows, including anaesthesia, were iteratively reviewed. Confidence ratings covered surgical skills and anaesthesia handling. Additional questions included demographics, academic level, prior experience and training background. Surveys were paper‐based, transcribed into Excel (Microsoft), and analysed using R (version 4.2.2, R Foundation for Statistical Computing). The study was reviewed by the University of Minnesota IRB (ID: STUDY00023791). The sample size reflected the number of students registered for the 10‐day campaign as a convenience sample.

### Objective Structured Assessment of Technical Skills evaluation

On the last day, supervising licensed veterinarians used an OSATS rubric aligned with ACVS teaching resources^17^ and tailored to the campaign context (Supporting Information S2) to rate each student on a 1–5 scale across five domains: tissue handling, time/motion, instrument use, surgical flow and procedural knowledge. Higher scores reflected better performance, with 1 indicating frequent errors or inadequate skill, and 5 representing mastery with minimal or no errors and smooth execution. Objective Structured Assessment of Technical Skills instruments are widely used across surgical training settings and have demonstrated acceptable reliability and construct validity,^18^, ^19^, ^20^, ^21^ supporting their use for formative assessment. Across the 10‐day campaign, supervising veterinarians directly observed each student during multiple sterilisation procedures in dogs and cats (ovariohysterectomy and orchiectomy). At the end of the campaign, OSATS scores were assigned for each student, summarising performance across all observed procedures. The rubric was refined through team and surgeon review for clarity and relevance. While no formal inter‐ or intra‐rater reliability training was conducted, all OSATS assessments were completed by surgical instructors familiar with the evaluation rubric. Self‐reported confidence and observer bias in OSATS are acknowledged limitations.^22^, ^23^

### Data analysis

We analysed pre–post‐differences in Likert‐scale confidence scores using the Wilcoxon signed‐rank test (two‐sided, *α* = 0.05), which is appropriate for paired ordinal data and small samples and does not assume normality. Effect sizes were computed as *r* = *Z*/√*N* (*N* = 13), where *Z* is the standardised Wilcoxon statistic and *N* is the number of paired observations, with 95% confidence intervals obtained by nonparametric bootstrap and magnitude labels using conventional cut points: small (0.10 to <0.30), medium (0.30 to <0.50) and large (≥0.50).^24^ For each domain we also summarised pre‐ and post‐intervention descriptive statistics (range, mean and standard deviation). Score distributions were visualised with boxplots. The OSATS outcomes were summarised descriptively (mean, standard deviation and range by domain) to characterise end‐of‐campaign competence.

## RESULTS

### Participants

Thirteen fourth‐year veterinary students (eight from Michigan State University and five from the University of Minnesota) participated. Across eight rural communities, 561 dog and cat sterilisations were performed (499 dogs and 62 cats). All the students completed both assessments with no exclusions. Across the 10‐day campaign, students participated in an average of approximately 26 surgeries per student as primary surgeons, with the remaining procedures performed by licensed, experienced veterinarians. Exploratory comparisons by school showed similar baseline confidence and similar pre–post‐gains across domains; no statistically significant between‐school differences were detected in change scores, and end‐of‐campaign OSATS means were comparable.

### Baseline characteristics

In the pre‐assessment survey, participants reported their surgical experience as part of their institution's official sterilisation training programme. Six students (46.2%) had performed more than 20 surgeries, two (15.4%) reported 6–10 surgeries and five (38.5%) had completed 1–5 procedures. Surgical experience outside formal curricula was common: 11 participants (84.6%) reported additional experience through externships, private clinics or volunteer settings, while only two (15.4%) had no outside experience. Five participants (38.5%) had previously taken part in sterilisation programmes in rural or low‐resource settings, while eight (61.5%) had not. Regarding satisfaction with their school's surgical curriculum, four participants (30.8%) were somewhat satisfied, two (15.4%) reported a neutral stance, two (15.4%) were somewhat dissatisfied and five (38.5%) were very dissatisfied.

### Analysis of confidence gains

**FIGURE 1:**
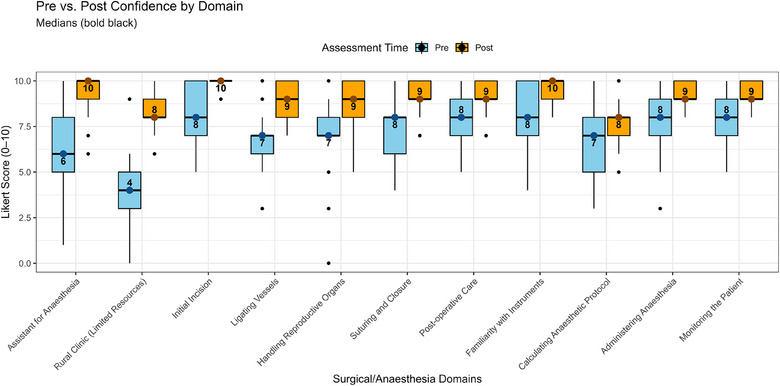
Boxplots illustrate the distribution of self‐reported confidence scores for each surgical domain before and after participation in the Mazunte Project campaign, with numeric medians annotated (bold black).

Pre‐ and post‐intervention assessments showed statistically significant increases in self‐reported confidence in 10 of 11 domains (Table 1 and Figure 1), with large effect sizes across all significant domains. The largest gains were observed for ligating blood vessels, handling reproductive organs and working in a rural clinic with limited resources; additional significant improvements were seen for making the initial incision, suturing and closing the incision, familiarity with surgical instruments, providing post‐operative care and monitoring, and intra‐operative patient monitoring. Confidence also increased in anaesthesia‐related domains (performing surgery while an assistant manages anaesthesia, administering anaesthesia for a sterilisation procedure, and monitoring the patient during a sterilisation procedure). Calculating injectable anaesthetic protocols was the only domain that did not show a statistically significant improvement (Table 1).

**TABLE 1:** Comparative pre‐ and post‐assessment confidence scores across surgical domains.

	Pre‐assessment				Post‐assessment							
Confidence domain	Range min	Range max	Mean	Standard deviation	Range min	Range max	Mean	Standard deviation	*p*‐Value	Effect size	95% CI (low, high)	Magnitude
With an assistant handling anaesthesia	1	10	6.31	2.53	6	10	9.15	1.34	0.006^*^	0.832	0.445, 1.000	Large
In a rural clinic with limited resources	0	9	3.92	2.29	6	10	8.23	1.09	0.002^*^	0.874	0.515, 1.000	Large
Making the initial incision	5	10	8.15	1.63	9	10	9.77	0.44	0.013^*^	0.765	0.351, 1.000	Large
Ligating blood vessels	3	10	6.54	2.03	7	10	8.92	1.04	0.002^*^	0.877	0.515, 1.000	Large
Handling reproductive organs	0	10	6.69	2.69	5	10	8.62	1.56	0.004^*^	0.861	0.482, 1.000	Large
Suturing and closing the incision	4	10	7.15	2.12	7	10	9.00	1.08	0.005^*^	0.836	0.445, 1.000	Large
Providing post‐operative care and monitoring	5	10	7.92	1.55	7	10	9.15	0.90	0.025^*^	0.633	0.046, 0.897	Large
Familiarity with surgical instruments	4	10	7.92	1.85	8	10	9.46	0.66	0.010^*^	0.735	0.192, 0.964	Large
Calculating appropriate injectable anaesthetic protocol	3	10	6.77	2.31	5	10	7.85	1.41	0.111	0.432	−0.206, 0.784	Moderate
Administering anaesthesia for an ovariohysterectomy/orchiectomy procedure	3	10	7.62	1.98	8	10	9.15	0.80	0.024^*^	0.618	−0.020, 0.887	Large
Monitoring the patient during an ovariohysterectomy/orchiectomy procedure	5	10	7.85	1.57	8	10	9.15	0.69	0.007^*^	0.809	0.402, 1.000	Large

*Note*: Table presents a comparative analysis of participants’ self‐reported confidence scores before and after their participation in the Mazunte Project surgical campaign.

* Statistically significant.

### Objective Structured Assessment of Technical Skills findings

End‐of‐campaign OSATS evaluations (Supporting Information S3) showed that most students performed within the upper range (scores 3–5) across all five domains. Instrument handling received the highest average score (mean = 3.94), followed by respect for tissue (mean = 3.84) and knowledge of specific procedure (mean = 3.81), indicating strong technical aptitude. Flow of operation (mean = 3.64) and time/motion (mean = 3.58) were slightly lower, reflecting occasional inefficiencies but overall satisfactory procedural rhythm. These results offer a snapshot of technical competence at the campaign's conclusion.

## DISCUSSION

This study evaluated whether participation in a high‐volume sterilisation campaign could enhance veterinary students’ self‐reported confidence and technical competence in surgical domains. Findings showed statistically significant increases in confidence across 10 of the 11 domains, with large effect sizes, suggesting improvement from immersive, hands‐on training. The greatest gains were in ligating blood vessels, working in rural clinics and handling reproductive organs. These skills are emphasised in high‐volume sterilisation settings.^25^

Calculating anaesthetic protocols was the only domain without statistically significant improvement, likely due to limited student involvement. This pattern is also consistent with the campaign's primary emphasis on surgical training, with anaesthesia‐related tasks treated as a secondary component of participation. In addition, anaesthesia competencies may be reinforced later in the veterinary curriculum through dedicated anaesthesia rotations or supervised clinical experiences; therefore, baseline confidence and the magnitude of change during a short field campaign may differ from domains that are intensively practiced during high‐volume surgery. Future iterations should incorporate a brief, structured dosing module and require each student to perform dose calculations during case intake; this integration would align assessment with practice and may strengthen gains in this domain.

Objective assessments using the OSATS rubric provided complementary insights into technical performance at the end of the campaign. Most students achieved high scores across the five evaluated domains, particularly in instrument handling and tissue respect. Time/motion and flow of operation scores were slightly lower but still reflected adequate surgical rhythm and decision making under supervision. Because OSATS was administered only post‐intervention, it provides a cross‐sectional snapshot rather than within‐student change. The overall pattern aligns with expected learning curves and underscores the educational value of repeated practice for skill development.^26^

These findings align with principles of deliberate practice in which improvement depends on focused goals, immediate feedback and repeated performance of representative tasks.^27^ The campaign's case volume, real‐time coaching and end‐of‐day debriefs created structured opportunities to refine tissue handling, haemostasis and operative flow, helping to explain the observed pre–post‐confidence gains and satisfactory end‐of‐campaign competence.^28^ The daily rhythm also mapped onto the experiential learning cycle, comprising concrete operative experience, guided reflection, consolidation of concepts and rapid reapplication in subsequent cases, which together likely enhanced learning efficiency.^13^, ^28^

Ovariohysterectomy and orchiectomy are among the most common small animal surgeries,^29^, ^30^, ^31^ and gaining competence in these techniques is a core educational goal for veterinary students. Prior studies have shown that students who perform or observe procedures such as orchiectomy report greater confidence than those without such exposure.^32^ Repeated practice not only improves familiarity with each surgical step but also builds muscle memory and self‐assurance, both key components of surgical proficiency.^32^ Although the procedures in this study were routine sterilisations, the underlying training mechanism of repeated performance with supervision and feedback parallels skill acquisition across many soft‐tissue surgeries, where foundational competencies such as tissue handling, haemostasis, instrument use and operative flow are transferable. Similar learning‐curve patterns and confidence gains have also been described in human surgical education, including structured skills courses or boot camps and simulation‐based training grounded in deliberate practice, reinforcing that repeated coached practice is a generalisable model for procedural learning.^26^, ^28^, ^33^

Consistent with previous findings, our results suggest that direct surgical experience may contribute to increased student confidence. Immersive training models such as the Mazunte Project offer valuable opportunities for students to develop technical skills in diverse, high‐volume clinical settings. Repeated exposure may have a cumulative effect on both competence and confidence. Future studies with larger sample sizes could explore this trend. Similar initiatives have been described in veterinary shelter medicine and community‐practice training programmes that integrate high‐quality, high‐volume spay and neuter experiences into the curriculum. For example, a shelter medicine surgery programme reported that students completed high case volumes and demonstrated improved efficiency with repeated procedures, supporting the educational value of concentrated repetition and feedback.^34^ Likewise, a shelter medicine externship study reported increased student self‐ratings for sterilisation procedures after the experience, consistent with confidence gains from hands‐on exposure.^35^

### Limitations

This study had several limitations. The small sample size (*n* = 13) and lack of a control group limit generalisability. Given the small convenience sample drawn from two US veterinary schools and a single field campaign, external validity is limited; findings should be considered preliminary and hypothesis‐generating rather than definitive.

Differences in prior surgical experience may have influenced confidence and performance. Self‐reported confidence may not reflect actual ability. In particular, metacognitive self‐assessment bias (over‐ or under‐estimation of one's competence) could have affected the reported confidence gains.^23^

The OSATS evaluations were only done at the end, with no formal rater reliability training, and possible observer bias. In addition, the absence of inter‐rater calibration means competence estimates should be interpreted cautiously given potential leniency/stringency and halo effects inherent to supervisor‐scored assessments. Future studies should incorporate baseline (pre‐intervention) OSATS, interim checks and post‐intervention assessments with standardised rater training.

Additionally, we did not systematically account for potential differences in school curricula, which may influence baseline confidence and learning trajectories; future studies should collect curriculum variables and stratify or adjust analyses accordingly. Learning gains may vary across institutions, supervision models, case‐mix and resource settings, underscoring the need for replication in larger, more diverse cohorts.

## CONCLUSION

Participation in the Mazunte Project was associated with a notable increase in veterinary students’ self‐reported confidence and satisfactory end‐of‐campaign technical competence. Although the intervention focused primarily on surgical education, it also contributed to One Health objectives by addressing animal health in underserved communities. This high‐volume, supervised service‐learning model may complement traditional curricula by providing concentrated operative experience, graduated responsibility and structured feedback in a real‐world setting, with skills that are likely transferable to other soft‐tissue procedures beyond routine sterilisation. Given the small, single‐campaign cohort and uncontrolled design, these findings should be interpreted cautiously. Larger, multi‐institution studies with baseline and follow‐up objective assessments and longer‐term tracking of independent surgical performance are needed to assess durability and generalisability.

## AUTHOR CONTRIBUTIONS

All the authors meet the ICMJE criteria for authorship, having made substantial contributions to the work and approving the final version for publication. Guillermo Arcega Castillo conceptualised the study, developed the data collection instruments, conducted the data analysis, created figures and tables and led the drafting of the manuscript. He contributed substantially to the interpretation of results and critical revisions for intellectual content and approved the final version of the manuscript. Rachael Schulte contributed to project coordination, provided input on the design of survey instruments and manuscript structure, and participated in data interpretation. She revised the manuscript critically for important intellectual content and approved the final version for publication. Melinda J. Wilkins provided mentorship and oversight throughout the study, including guidance on IRB protocol development, refinement of the study methodology and interpretation of findings. She contributed to critical revisions of the manuscript for intellectual content and approved the final version for publication.

## CONFLICTS OF INTEREST

The authors declare they have no conflicts of interest.

## FUNDING INFORMATION

The authors received no specific funding for this work.

## ETHICS STATEMENT

The study was reviewed by the University of Minnesota Institutional Review Board (ID: STUDY00023791).

## Supporting information



Supporting Information

Supporting Information

Supporting Information

## Data Availability

Data supporting this study's findings, including additional materials, are available from the corresponding author upon request.
