# Supplementary material for: Surgical confidence and competence among US veterinary students after a high‐volume sterilisation campaign in rural Mexico
Source: Vet Rec Open. 2026 Feb 15;13(1):e70025. doi: 10.1002/vro2.70025 (PMC12906661; doi:10.1002/vro2.70025)
Supplement: Supplementary file 1 — Supporting Information [file VRO2-13-e70025-s002.pdf]

Please provide your first name and the initial of your last name: ____________________
What is the name of the university where you are currently pursuing or have completed your veterinary medicine education? ___________________________________

Section A: Background Experience
1. Have you had any hands-on experience with spay/neuter surgeries prior to this course? (Select one option)

- ☐ Yes, I have independently performed a surgery.
- ☐ Yes, I have assisted during a surgery.
- ☐ Yes, I have observed a surgery without participating.
- ☐ No, I have no prior experience with spay/neuter surgeries.

2. Please select the option that best reflects the number of surgeries you performed as the surgeon during your time as a veterinary student: (only include surgeries that were part of your school's official program)

- ☐ None; I did not perform any surgeries as the surgeon.
- ☐ 1–5 surgeries as the surgeon.
- ☐ 6–10 surgeries as the surgeon.
- ☐ 11–20 surgeries as the surgeon.
- ☐ More than 20 surgeries as the surgeon.

2-1. If you have had surgical experience outside your school's official program (e.g., private clinics, externships), please describe briefly here:_________________________
3. How do you feel about the veterinary surgical curriculum at your school, specifically regarding spay/neuter training? (Select one option)

- ☐ Very satisfied; I feel it provides comprehensive and adequate training.
- ☐ Somewhat satisfied; it covers the basics but could use more hands-on opportunities.
- ☐ Neutral; I have no strong opinion about the surgical curriculum.
- ☐ Somewhat dissatisfied; it lacks sufficient practical experience or preparation.
- ☐ Very dissatisfied; it does not adequately prepare students for real-world surgical procedures.

Section B: Confidence in Specific Scenarios Rate your confidence level in performing a spay/neuter surgery under the following scenarios:
4. With an assistant handling anesthesia and monitoring. (0-10): ______
5. In a rural clinic with limited resources. (0-10): ______
6. Making the initial incision. (0-10): ______
7. Ligating blood vessels. (0-10): ______
8. Handling reproductive organs (e.g., ovaries, testicles). (0-10): ______
9. Suturing and closing the incision. (0-10): ______
10. Providing post-operative care and monitoring. (0-10): ______
Section D: Knowledge and Familiarity with Surgical Instruments
11. How confident are you with using the surgical instruments needed for spay/neuter procedures? (0-10): ______
Section F: Experience in Rural or Resource-Limited Areas
12. Have you participated in spay/neuter programs in rural or resource-limited settings? (Select one option)

- ☐ Yes, I have participated in one or more programs. If yes, how did this experience impact your confidence in performing surgeries in similar settings? Impact on Confidence (0 = no impact, 10 = very significant impact): ____
- ☐ No, I have not participated in such programs.

Section G: Confidence Improvement Preferences
13. Which of the following would most help improve your confidence in performing spay/neuter surgeries? (Select up to two options)

- ☐ More hands-on practice under supervision.
- ☐ Additional theoretical knowledge on surgical procedures.
- ☐ More opportunities to observe surgeries.
- ☐ Feedback and guidance from senior veterinarians.
- ☐ Other (please specify): ____

Section H: Confidence in Anesthesia and Monitoring
14. How confident are you in calculating an appropriate injectable anesthetic protocol using the medications available? (0-10): ______
15. How confident are you in administering anesthesia for a spay/neuter procedure? (0-10): ___
16. How confident are you in monitoring the patient during a spay/neuter procedure? (0-10): ___
Section J: Communication with Pet Owners
17. How prepared do you feel to educate pet owners on post-operative care and address their concerns? (This question refers to your general preparedness and is not specific to the Mazunte Project)

- ☐ Very prepared; confident addressing most questions.
- ☐ Somewhat prepared; comfortable with basic guidance.
- ☐ Not very prepared; would require support in communication.
- ☐ Not prepared at all; need additional training in post-operative guidance.

Section K: Comments
18. Do you have any additional comments or suggestions regarding your experience with surgical training during veterinary school, this assessment, and the "Mazunte Project"?

____
