# Supplementary material for: Surgical confidence and competence among US veterinary students after a high‐volume sterilisation campaign in rural Mexico
Source: Vet Rec Open. 2026 Feb 15;13(1):e70025. doi: 10.1002/vro2.70025 (PMC12906661; doi:10.1002/vro2.70025)
Supplement: Supplementary file 2 — Supporting Information [file VRO2-13-e70025-s003.docx]

OSATS (Objective Structured Assessment of Technical Skills)

OSATS (Objective Structured Assessment of Technical Skills)
is a structured method used to evaluate practical surgical skills in a standardized and objective way.

- Student/Trainee: __________________________________________________
- Supervisor: __________________________________________________
- Date: __________________________________________________

GLOBAL RATING SCALE OF OPERATIVE PERFORMANCE
Please write the number corresponding to the candidate’s performance for each category irrespective of training level.

Skill #1

|  | 1  Frequently used unnecessary force on tissue or caused damage by inappropriate use of instruments. | 2 | 3 Handled tissue carefully but occasionally caused inadvertent damage. | 4 | 5 Consistently handled tissue appropriately with minimal damage. | NA |
| --- | --- | --- | --- | --- | --- | --- |
| **Respect for Tissue Score** |  |  |  |  |  |  |

Skill #2

|  | 1  Made many unnecessary moves. | 2 | 3 Demonstrated efficient time/motion but included some unnecessary moves. | 4 | 5 Showed clear economy of movement and maximum efficiency. | NA |
| --- | --- | --- | --- | --- | --- | --- |
| **Time and Motion** |  |  |  |  |  |  |

Skill #3

|  | 1  Repeatedly made tentative or awkward moves with instruments due to inappropriate use. | 2 | 3 Demonstrated competent use of instruments but occasionally appeared stiff or awkward. | 4 | 5 Made fluid moves with instruments and showed no awkwardness. | NA |
| --- | --- | --- | --- | --- | --- | --- |
| **Instrument Handling** |  |  |  |  |  |  |

Skill #4

|  | 1  Frequently stopped operating and seemed unsure of the next move. | 2 | 3 Demonstrated some forward planning with reasonable progression of the procedure. | 4 | 5 Clearly planned the course of the operation with an effortless flow from one move to the next. | NA |
| --- | --- | --- | --- | --- | --- | --- |
| **Flow of Operation** |  |  |  |  |  |  |

Skill #5

|  | 1  Demonstrated deficient knowledge and needed specific instruction at most steps. | 2 | 3 Knew all important steps of the operation. | 4 | 5 Demonstrated familiarity with all aspects of the operation. | NA |
| --- | --- | --- | --- | --- | --- | --- |
| **Knowledge of Specific Procedure** |  |  |  |  |  |  |

Comments from the Supervising Surgeon:

________________________________________________________________
